# Supplementary material for: Reactivated endogenous retroviruses promote protein aggregate spreading
Source: Nat Commun. 2023 Aug 18;14:5034. doi: 10.1038/s41467-023-40632-z (PMC10439213; doi:10.1038/s41467-023-40632-z)

SUPPLEMENTARY MATERIAL

Reactivated endogenous retroviruses promote protein aggregate spreading

Shu Liu[1, 6, 7], Stefanie-Elisabeth Heumüller[1, 7], André Hossinger[1], Stephan A. Müller[2], Oleksandra Buravlova[1], Stefan F. Lichtenthaler[2, 3, 4], Philip Denner[1] and Ina M. Vorberg[1, 5**]

[1]*German Center for Neurodegenerative Diseases Bonn (DZNE), Venusberg Campus 1/ 99, 53127 Bonn, Germany*
[2]*German Center for Neurodegenerative Diseases (DZNE), Munich, Germany*
[3]*Neuroproteomics, School of Medicine, Klinikum rechts der Isar, Technical University of Munich, 81675 Munich, Germany*
[4]*Munich Cluster for Systems Neurology (SyNergy), Munich, Germany*
[5]*Department of Neurology, Rheinische Friedrich-Wilhelms-Universität Bonn, Germany Sigmund-Freud Str. 25, 53127 Bonn, Germany*
[6]*Present address: German Federal Institute for Risk Assessment (BfR), German Centre for the Protection of Laboratory Animals (Bf3R), Max-Dohrn-Straße 8-10, 10589 Berlin, Germany*
[7]*These authors contributed equally: Shu Liu, Stefanie-Elisabeth Heumüller*

**Corresponding author: ina.vorberg@dzne.de
Keywords: Prion-like, Tau, Sup35, extracellular vesicle, endogenous retrovirus, murine leukemia virus, human endogenous retrovirus, HERV, Env

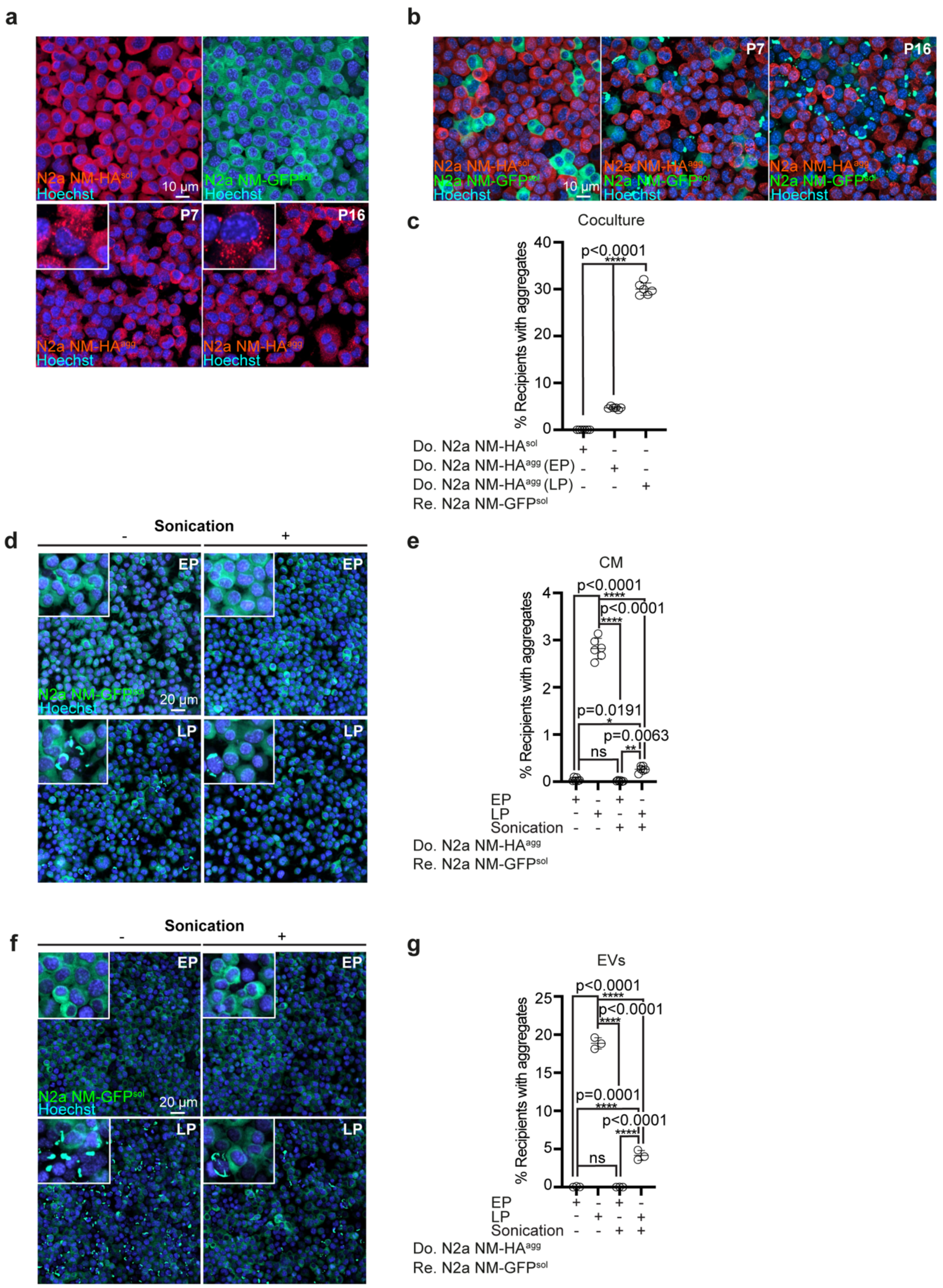

Supplementary Fig. 1. Increased aggregate induction by donors that have been in culture for prolonged time. a. N2a NM cell culture model. N2a cells expressing soluble NM-HA (NM-HA^{sol}, upper left image) or soluble NM-GFP (NM-GFP^{sol}, upper right). N2a NM-HA^{sol} cells were exposed to recombinant NM fibrils and donor clone N2a NM-HA^{agg} s2E was established that consistently produces NM-HA aggregates (NM-HA^{agg}, lower images). For

simplicity, we call the clone N2a NM-HA^{agg}. Shown are early (P7) and late passages of the clone (P16). Insets show close-ups. **b.** Cocultures of N2a NM-GFP^{sol} with N2a NM-HA^{agg} cells of early and late passage. As controls, recipients were also cocultured with N2a NM-HA^{sol} cells. NM-HA was detected by anti-HA antibodies. **c.** Cryopreserved N2a NM-HA^{agg} cells of passage 21 retain their NM aggregate high inducing activity. Donor cells had also been frozen at passage 1. Donors with different passaging histories were defrosted and cultured for less than 6 passages (thereafter termed "EP" for "early passage" and "LP" for "late passage", respectively). Donors were cocultured with N2a NM-GFP^{sol} cells and recipients with NM-GFP^{agg} were detected 16 h later. As control, recipients were cultured with N2a NM-HA^{sol} cells. **d.** Confocal images of recipient cells exposed to conditioned medium from donors of early or late passage. To destroy vesicles, medium was sonicated. Insets show close-ups. **e.** Quantitative analysis of aggregate induction by conditioned medium shown in **(d)**. **f.** Confocal images of recipient cells exposed to isolated EVs from donors of early or late passage. **g.** Quantitative analysis of aggregate induction by EVs. To destroy vesicles, EVs were sonicated. All data are shown as the means ± SD from three (g) or six (c, e) replicate cell cultures. Three (c, e, g) independent experiments were carried out with similar results. P-values calculated by one-way ANOVA with Tukey´s multiple comparisons. Source data are provided as a Source Data file.

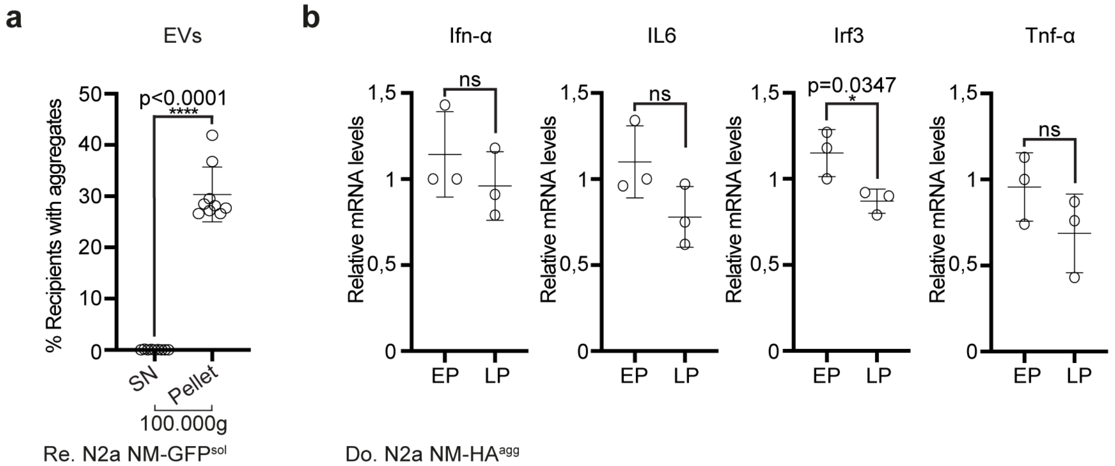

Supplementary Fig. 2. Cytokine involvement in intercellular protein aggregate induction. **a.** No seeding activity in conditioned medium following EV depletion by ultracentrifugation. **b.** Cytokine mRNA levels in early (EP) and late passage (LP) N2a NM-HA^{agg} cells. All data are shown as the means ± SD from nine (a) or three (b) replicate cell cultures. Three (a, b) independent experiments were carried out with similar results. P-values calculated by two-tailed unpaired Student´s t-test (a, b). Source data are provided as a Source Data file.

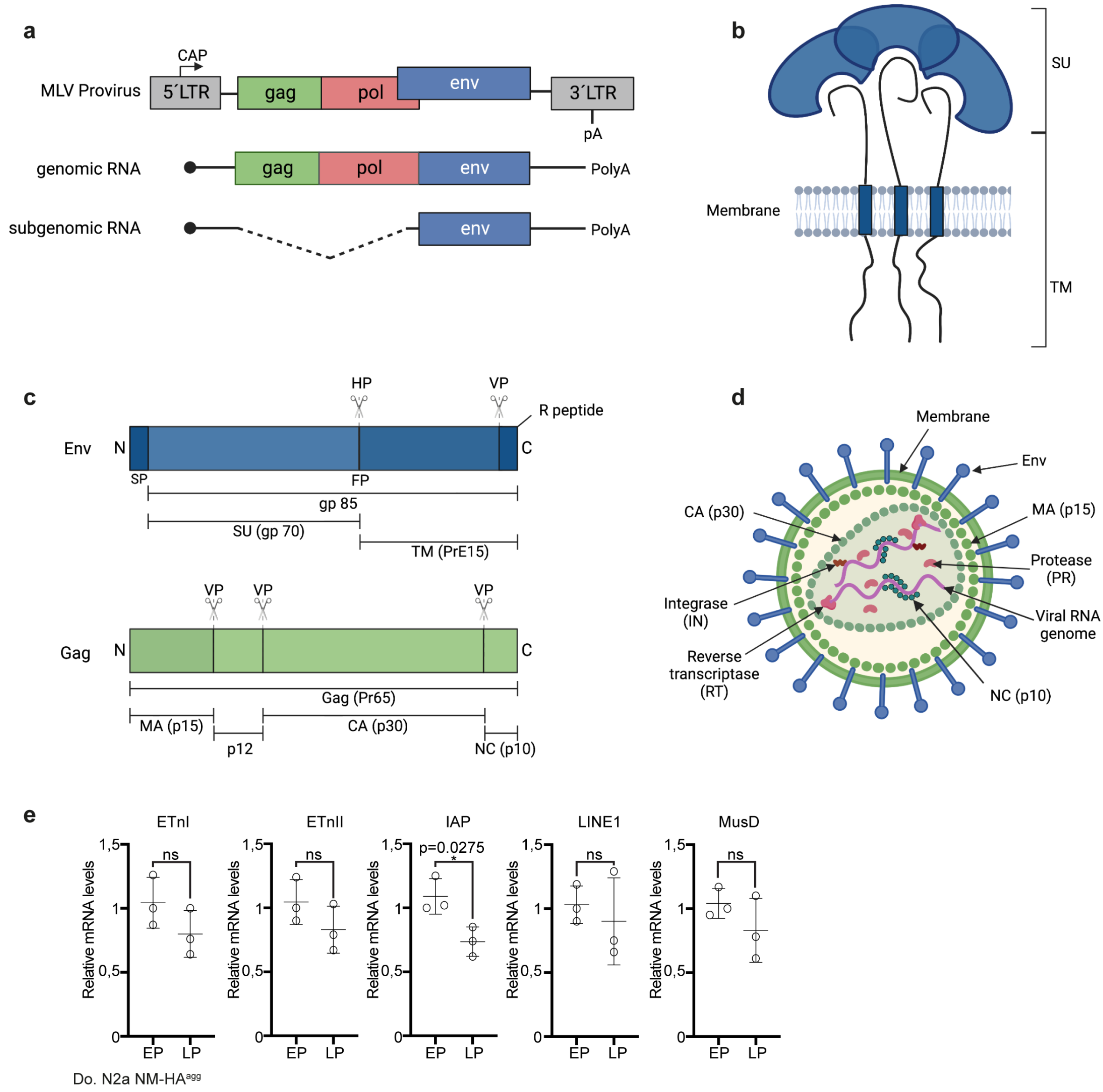

Supplementary Fig. 3. Genomic and structural organization of MLV. a. Genome organization of endogenous and exogenous MLV provirus and transcripts. The positive strand genomic RNA contains a 5’ long terminal repeat (LTR), a 5’ leader sequence, followed by the open reading frames coding for Gag, Pol, Env, and a 3’ LTR. Transcription of a single full-length mRNA is initiated in the 5’ LTR. Splicing of this mRNA generates the subgenomic *env* mRNA. Gag and Pol polyproteins are translated from full-length mRNA, Env is translated from the subgenomic mRNA. **b**. The viral ligand for receptor binding is composed of Env trimers. Surface unit (SU) and transmembrane unit (TM) are linked by an intersubunit disulfide bond. Cleavage of a carboxyterminal R peptide in TM by the viral protease activates Env and results in membrane fusion. **c.** Processing of Env and Gag polyproteins. Host furin protease (HP) cleaves Env into SU and TM, the viral protease (VP) activates Env by removing the R peptide. The Gag polyprotein is cleaved by the viral protease into subunits such as matrix (MA), capsid (CA) and nucleocapsid (NC). Inhibition of VP prevents maturation of Gag and Env. SP: Signal peptide. FP: Furin cleavage site. **d.** MLV particle. Two copies of the genomic RNAs associated with the nucleocapsid are packaged into the capsid, which also contains viral enzymes. The capsid is surrounded by a matrix and a membrane, decorated by Env. Panels a-d were created with Biorender.com. **e.** Transcript levels of murine retroelements IAP, MusD, EtnI/II and LINE-1 remain largely unaltered upon prolonged cell culture. All data are shown as the means

± SD from three replicate cell cultures. Three independent experiments were carried out with similar results. P-values calculated by two-tailed unpaired Student´s t-test (e). ns: non-significant. Source data are provided as a Source Data file.

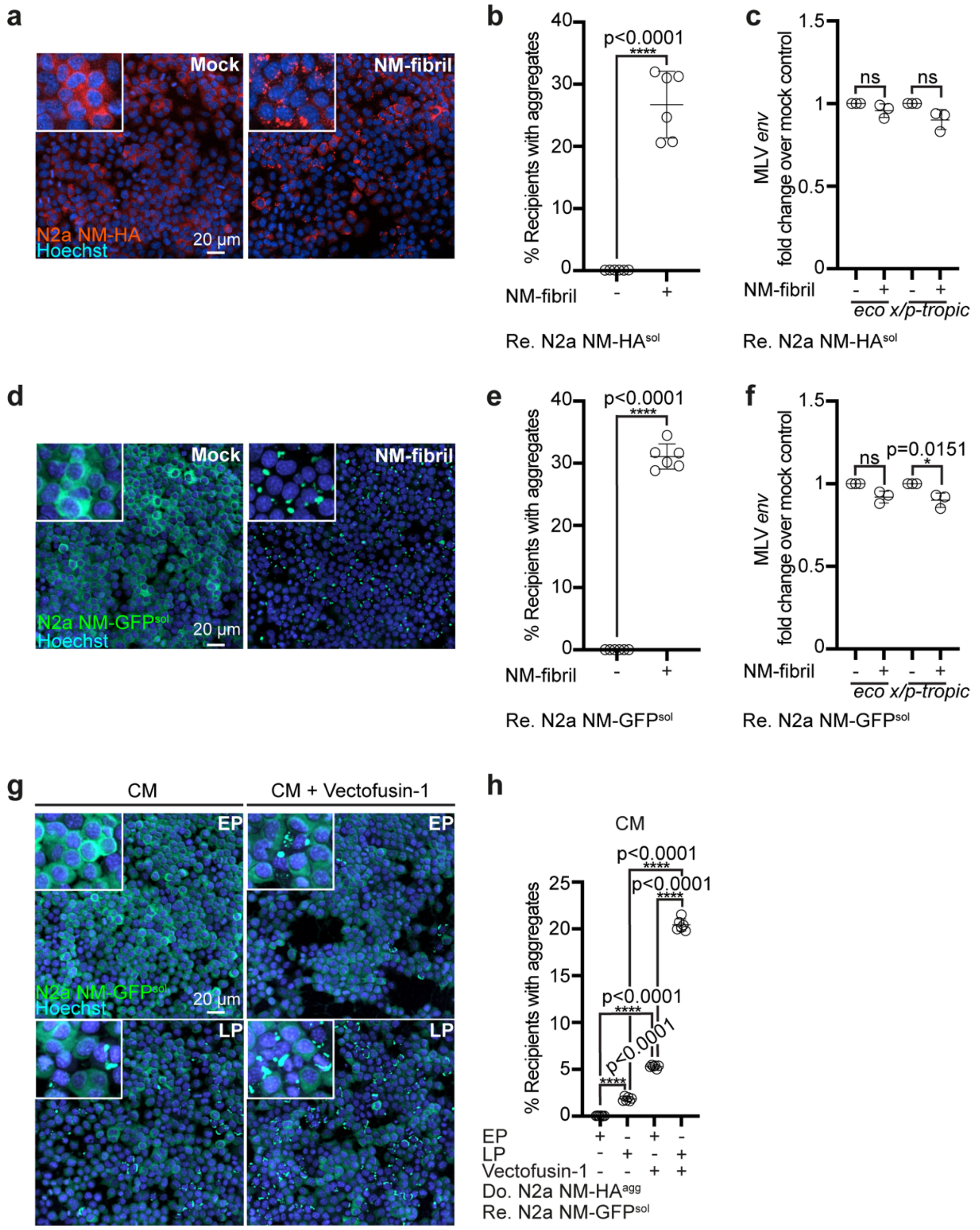

Supplementary Fig. 4. Upregulation of endogenous retrovirus is not caused by aggregate induction. a. N2a NM-HA^{sol} cells were exposed to 1 µM recombinant NM fibrils (monomer equivalent) and cells were subsequently cultured for 15 days. Untreated cells served as controls. NM-HA was detected using anti-HA antibody. Insets show close-ups. **b.** Quantitative analysis of recipient cells with induced aggregates. NM-HA aggregate induction was monitored 72 h post exposure. **c.** Expression of eco- and xeno-/polytropic X/P-MLV was detected by RT-PCR. **d.** N2a NM-GFP^{sol} cells were exposed to 1 µM recombinant NM fibrils (monomer equivalent) and cells were subsequently cultured for 15 days. Untreated cells served as controls. Insets show close-ups. **e.** Quantitative analysis of recipient cells with induced aggregates. NM-GFP aggregate induction was monitored 72 h post exposure. **f.** Expression of eco- and X/P-tropic MLV was detected by RT-PCR. **g.** Recipient N2a NM-GFP^{sol} cells were exposed to conditioned medium (CM) from early or late passage donors in the presence or absence of Vectofusin-1. Aggregate induction was assessed 16 h later. **h.** Quantitative analysis of **(g)**. All

data are shown as the means ± SD from three (c, f) or six (b, e, h) replicate cell cultures. Three (b, c, e, f, h) independent experiments were carried out with similar results. P-values calculated by two-tailed unpaired Student´s t-test (b, e), one-way ANOVA with Tukey´s multiple comparisons (h) or one-way ANOVA with Bonferroni´s multiple comparisons (c, f). ns: non-significant. Source data are provided as a Source Data file.

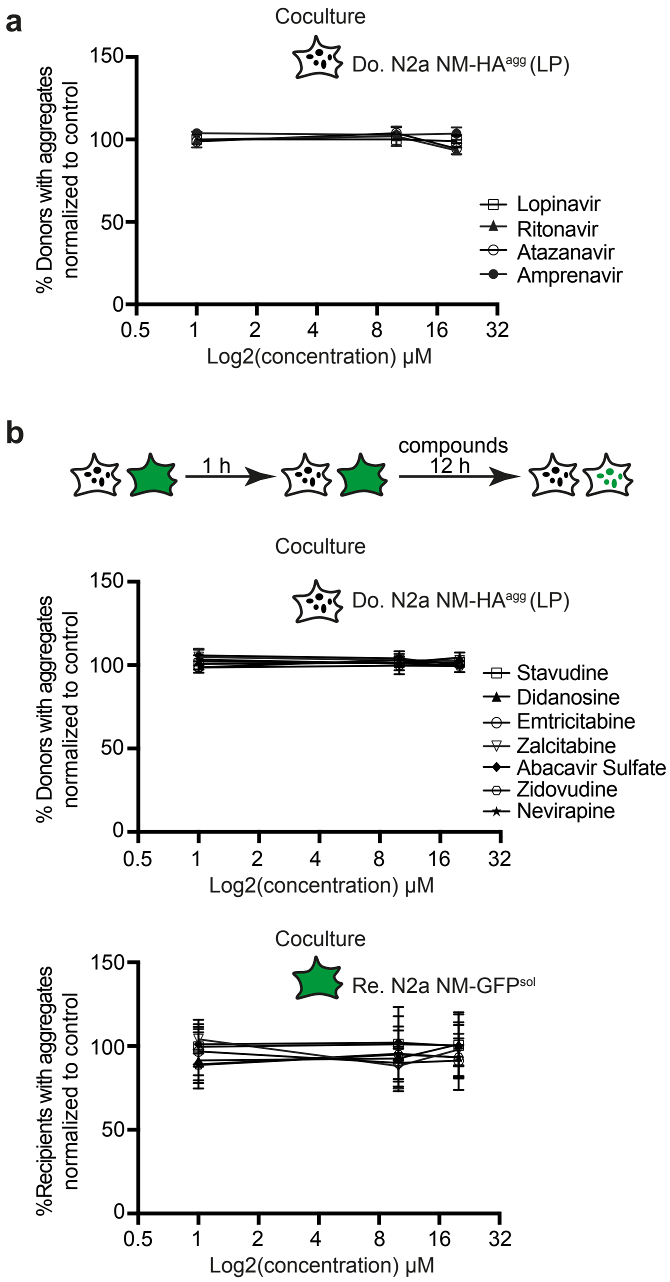

Supplementary Fig. 5. HIV integrase and reverse transcriptase inhibitors and HCV protease inhibitors fail to inhibit intercellular aggregate induction. a. Effect of HIV protease inhibitors on preexisting NM-HA aggregates in donor cells. Shown are the percentages of donor cells with aggregates upon exposure to different concentrations of drugs. **b.** N2a NM-HA^{agg} and N2a NM-GFP^{sol} cells were cocultured in the presence of HIV or HCV inhibitors. Effect of different concentrations of inhibitors on the percentage of donor and recipient cells harboring NM-HA^{agg} or NM-GFP^{agg}, respectively. % cells with aggregates were normalized to control cocultures treated with DMSO. All data are shown as the means ± SD from three (a, b) replicate cell cultures. Three (a, b) independent experiments were carried out with similar results. Source data are provided as a Source Data file.

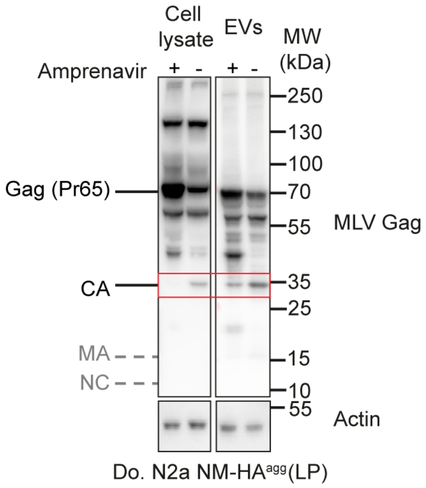

Supplementary Fig. 6. Amprenavir inhibits maturation of MLV proteins in cells and EVs. Donor cells were preincubated with Amprenavir for 72 h. Medium was exchanged for EV-depleted medium. EVs and cells were harvested after 3 days and lysates were assessed for MLV Gag maturation by Western blot using mouse anti-MLV Gag ab100970. This antibody is directed against the capsid (CA) and detects CA and its precursors, but not the nucleocapsid (NC) or p12. Expected heights of these proteins are indicated in grey. The red box indicates changes in capsid maturation. This Western blot has been run once.

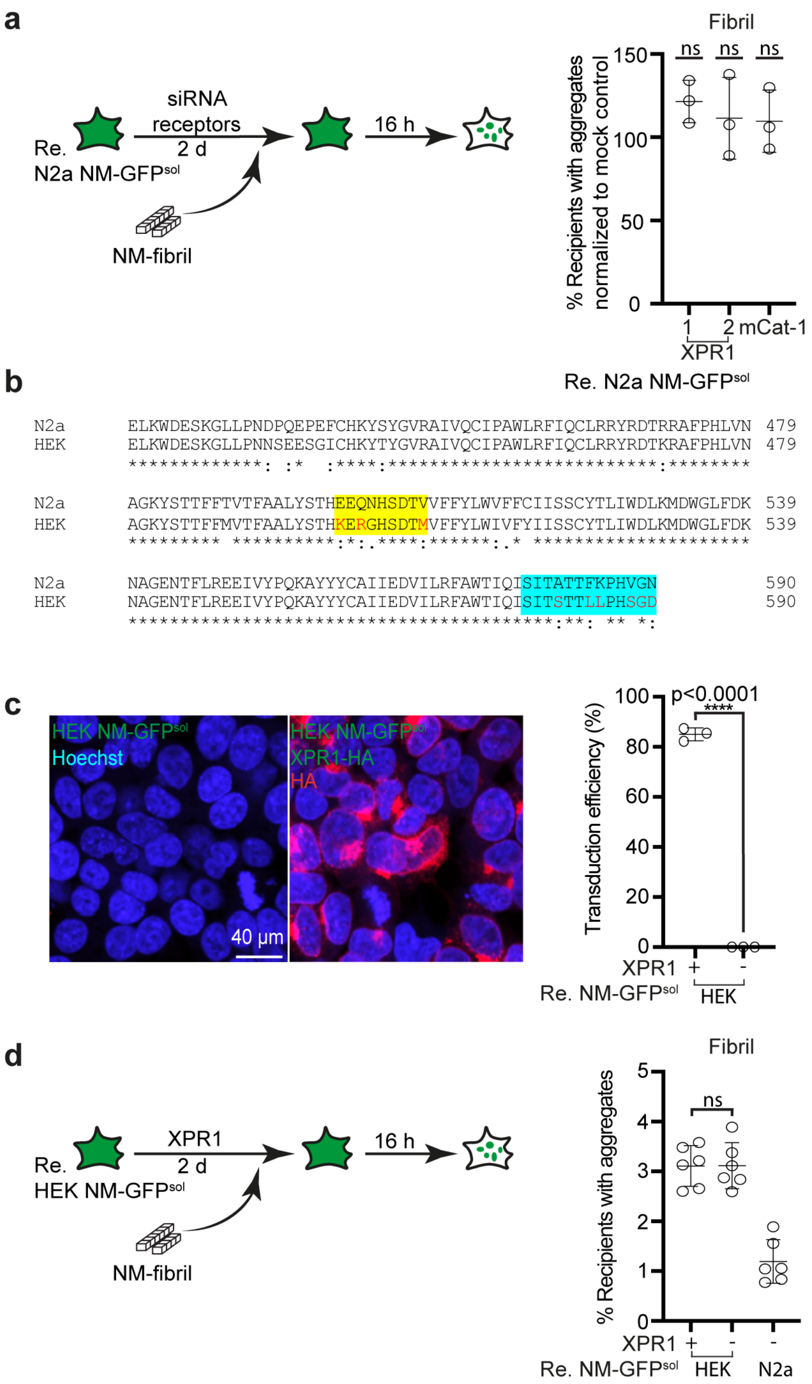

Supplementary Fig. 7. N2a XPR1 mediates intercellular protein aggregate dissemination. **a.** Recipient N2a NM-GFP^{sol} cells were transfected with two siRNAs against the XPR1 receptor or one siRNA against mCat-1. As controls, recipients were transfected with non-silencing siRNA. Two days later, recipients were exposed to 10 µm recombinant NM fibril (monomer equivalent). Recipients with NM-GFP^{agg} were quantified 16 h post exposure. **b.** Alignment of the amino acid sequences of N2a and HEK XPR1 ECL3 and ECL4 domains critical for viral receptor tropism. Shown is the sequence deduced from the analyzed nucleotide region. ECL3 and ECL4 domains are symbolized by yellow and blue boxes, respectively. Sequence variations in ECL3 (three polymorphisms) and ECL4 (6 polymorphisms) are shown in red. **c.** HEK NM-GFP^{sol} cells stably expressing the HA epitope-tagged N2a XPR1 variant. Cells were transfected with a Piggyback vector coding for the transgene. A XPR1-positive clone was subsequently selected upon puromycin treatment. Shown are HEK cells with and

without XPR1-HA expression detected by anti-HA antibody. At least 200 cells in three independent images were analyzed for XPR1-HA expression, demonstrating receptor expression in 85,5 ± 2,6 % cells. **d.** Recipient HEK NM-GFP^{sol} cells were transfected with a plasmid coding for the N2a XPR1 receptor or empty plasmid for 2 days. Afterwards, pretreated recipients were exposed to 10 µm recombinant NM fibril (monomer equivalent). As controls, recipients N2a NM-GFP^{sol} were induced with NM fibril the same way. Recipients with NM-GFP^{agg} were quantified 16 h post exposure. All data are shown as the means ± SD from three (a, c) or six (d) replicate cell cultures. Three (a, c, d) independent experiments were carried out with similar results. P-values calculated by two-tailed unpaired Student´s t-test (c), one-way ANOVA with Dunnett´s post hoc test (a) or one-way ANOVA with Bonferroni´s multiple comparisons (d). ns: non-significant. Source data are provided as a Source Data file.

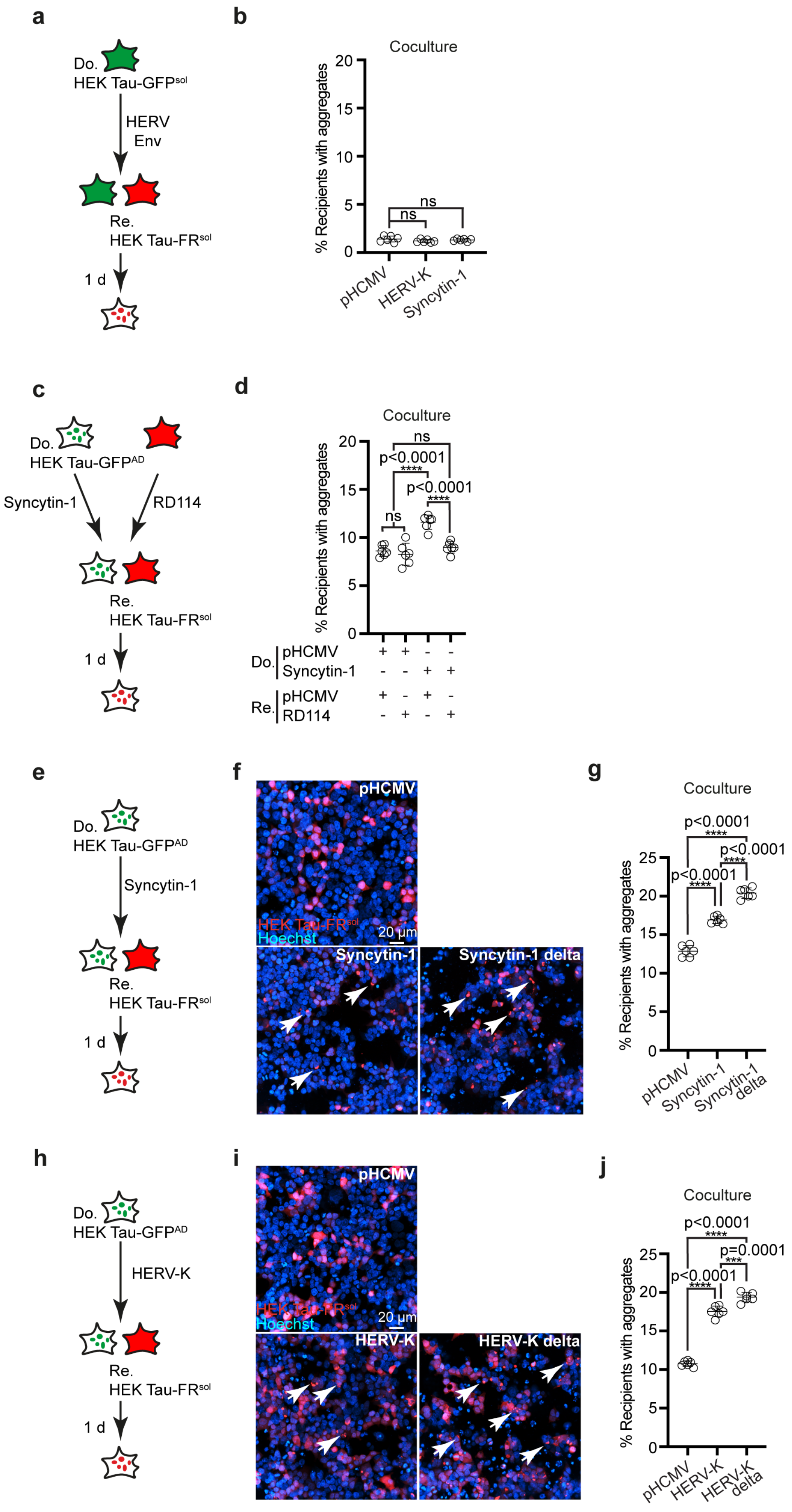

a
Do.
HEK Tau-GFPsol
HERV Env
Re.
HEK Tau-FRsol
1 d
b
Coculture
% Recipients with aggregates
ns
ns
pHCMV
HERV-K
Syncytin-1
c
Do.
HEK Tau-GFPAD
Syncytin-1
RD114
Re.
HEK Tau-FRsol
1 d
d
Coculture
% Recipients with aggregates
ns
p<0.0001
p<0.0001
ns
Do.
pHCMV
Syncytin-1
Re.
pHCMV
RD114
e
Do.
HEK Tau-GFPAD
Syncytin-1
Re.
HEK Tau-FRsol
1 d
f
pHCMV
HEK Tau-FRsol
Hoechst
20 μm
Syncytin-1
Syncytin-1 delta
g
Coculture
p<0.0001
p<0.0001
p<0.0001
% Recipients with aggregates
pHCMV
Syncytin-1
Syncytin-1 delta
h
Do.
HEK Tau-GFPAD
HERV-K
Re.
HEK Tau-FRsol
1 d
i
pHCMV
HEK Tau-FRsol
Hoechst
20 μm
HERV-K
HERV-K delta
j
Coculture
p<0.0001
p=0.0001
p<0.0001
% Recipients with aggregates
pHCMV
HERV-K
HERV-K delta

Supplementary Fig. 8. Receptor-ligand interactions of HERV-K Env and HERV-W Syncytin-1 contribute to Tau aggregate spreading. a. Donor cells expressing soluble Tau-GFP were transfected with empty vector pHCMV or vectors coding for V5 epitope-tagged envelope proteins of HERV-K and -W. Donors were subsequently cocultured with recipient cells to assess the effect of viral proteins on spontaneous protein aggregation. **b**. No spontaneous induction of protein aggregation by expression of HERV envelope proteins. **c.** Donor cells expressing Syncytin-1 or mock-transfected donors were cocultured with mock-transfected or RD114 Env expressing recipients. RD114 Env uses the same receptor as Syncytin-1 and can be used to block the receptor. **d.** Quantitative analysis of recipients with Tau-FR aggregates. RD114 in recipients blocks the receptor for Syncytin-1, thereby neutralizing the effect of Syncytin-1 on aggregate spreading. **e.** Experimental design. HEK Tau-GFP^{AD} cells were transfected with empty plasmid, plasmid coding for Syncytin-1 or its deletion mutant Syncytin-1 delta. Donor cells were subsequently cocultured with recipient HEK Tau-FR^{sol} cells. **f.** Representative images of cocultures. Arrowheads mark Tau-FR aggregates. Note that we have not imaged for Tau-GFP. **g.** Quantitative analysis of the percentage of recipient cells with induced Tau-FR aggregates. **h.** Experimental design. HEK Tau-GFP^{AD} cells were transfected with empty plasmid, plasmid coding for HERV-K Env or its deletion mutant HERV-K delta. Donor cells were subsequently cocultured with recipient HEK Tau-FR^{sol} cells. **i.** Representative images of cocultures. Arrowheads mark Tau-FR aggregates. Note that we have not imaged for Tau-GFP. **j.** Quantitative analysis of the percentage of recipient cells with induced Tau-FR aggregates. All data are shown as the means ± SD from six replicate cell cultures. Three independent experiments were carried out with similar results. P-values calculated by one-way ANOVA with Dunnett´s post hoc test (b) or one-way ANOVA with Tukey´s multiple comparisons (d, g, j). ns: non-significant. Source data are provided as a Source Data file.

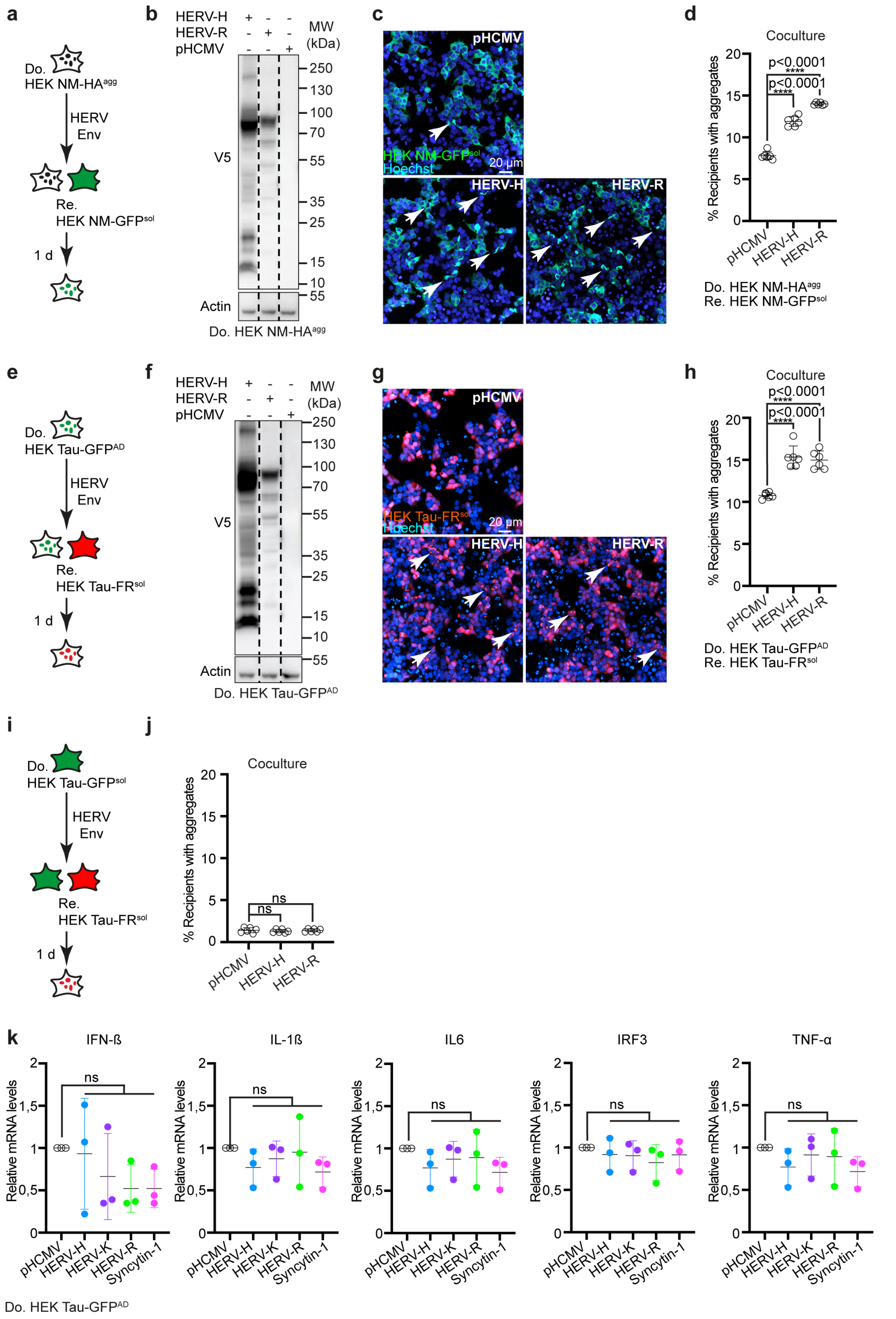

Supplementary Fig. 9. Envelope proteins from different HERV clades increase intercellular protein aggregate spreading. a. Experimental workflow. Donor HEK cells

stably propagating aggregated NM-HA were transfected with plasmids coding for V5-epitope tagged HERV-R or -H Envs. Cells were subsequently cocultured with recipient HEK NM-GFP^{sol}. **b.** Western blot analysis of donor clone transfected with plasmids. Additional lanes were excised for presentation purposes (dashed lines). Samples were run twice on different membranes for detection of Actin. **c.** Coculture of donor and recipient HEK cells (a). Note that we have not stained the donors in this experiment. **d.** Quantitative analysis of the percentage of recipient cells with induced aggregates upon coculture. **e.** Tau-GFP^{AD} were transfected with plasmid coding for V5 epitope-tagged HERV -R and -H Envs and cells were subsequently cocultured with recipient HEK cells expressing Tau-FR^{sol}. **f.** Western blot analysis of donor clone (e) transfected with plasmids coding for HERV Envs. Additional lanes were excised (dashed lines). **g.** Coculture of donor and recipient cells. **h.** Quantitative analysis of the percentage of recipient cells with induced aggregates. **i.** Donor cells expressing soluble Tau-GFP were transfected with empty vector pHCMV or vectors coding for V5 epitope-tagged envelope proteins of HERV-R and -H clades. Donors were subsequently cocultured with recipient cells to assess the effect of viral proteins on spontaneous protein aggregation. **j**. No spontaneous induction of protein aggregation by expression of HERV-R and -H envelope proteins. **k.** Inflammatory cytokine transcripts in transfected donor HEK Tau-GFP^{AD} cells, assessed by real-time PCR. Samples were run on different assay plates and normalized to pHCMV controls set to 100 %. All data are shown as the means ± SD from six (d, h, j) or three (k) replicate cell cultures. Three (d, h, j, k) independent experiments were carried out with similar results. P-values calculated by one-way ANOVA with Dunnett´s post hoc test. ns: non-significant. Source data are provided as a Source Data file.

Supplementary Figure 6: Uncropped scan of gels for main figures

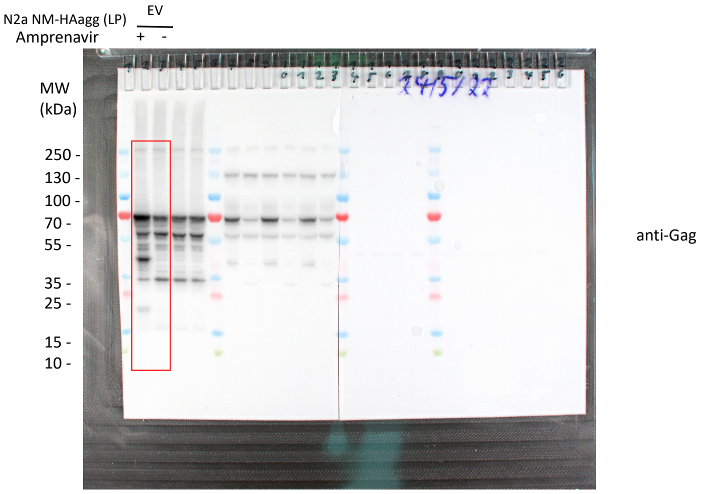

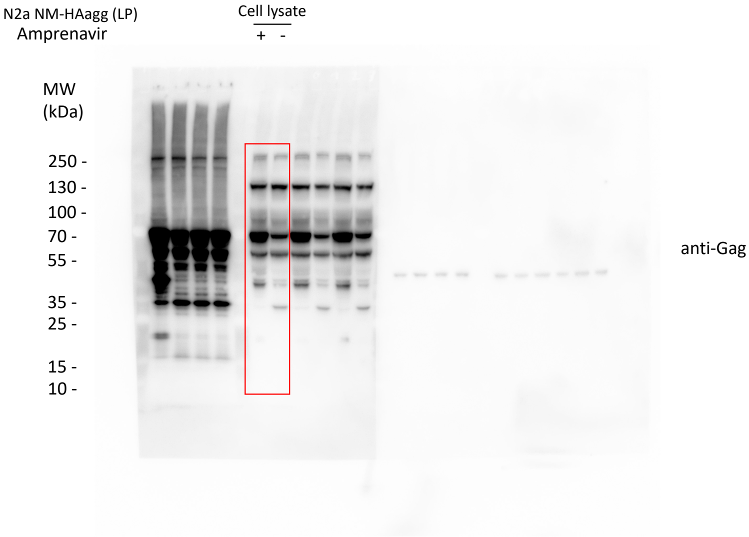

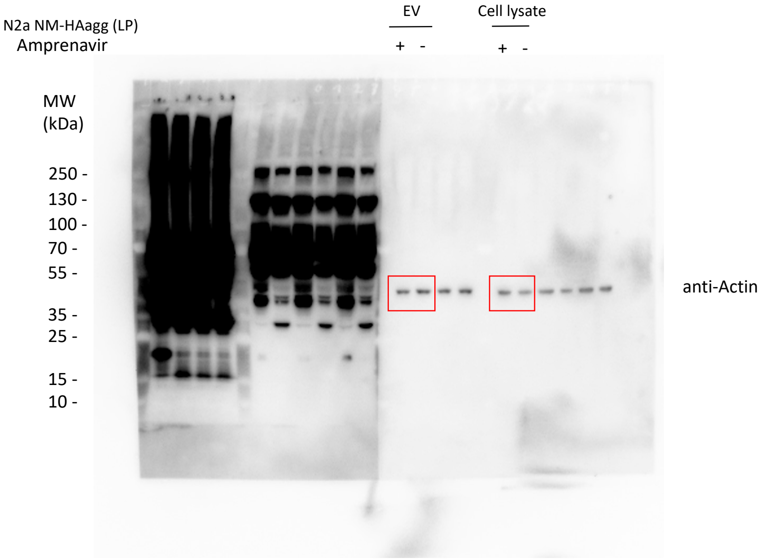

Supplementary Figure 9: Uncropped scan of gels for main figures

Supplementary Fig. 9b
Do. HEK NM-HAagg

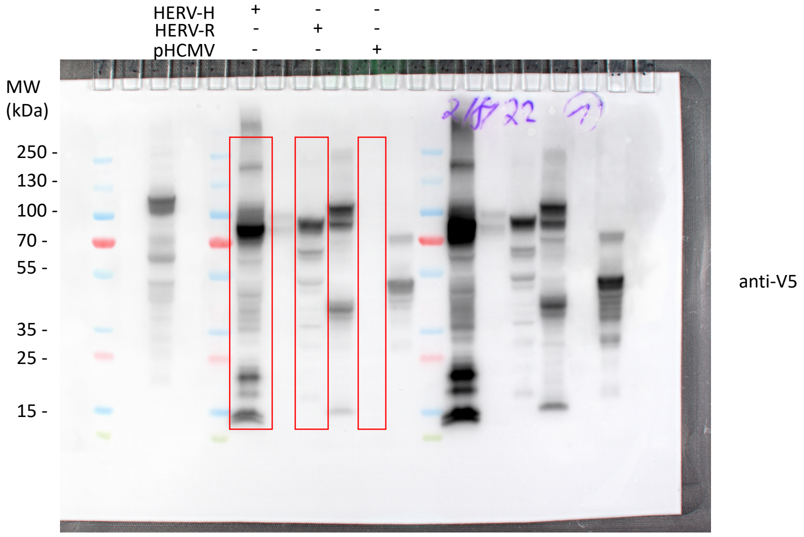

Supplementary Fig. 9b
Do. HEK NM-HAagg

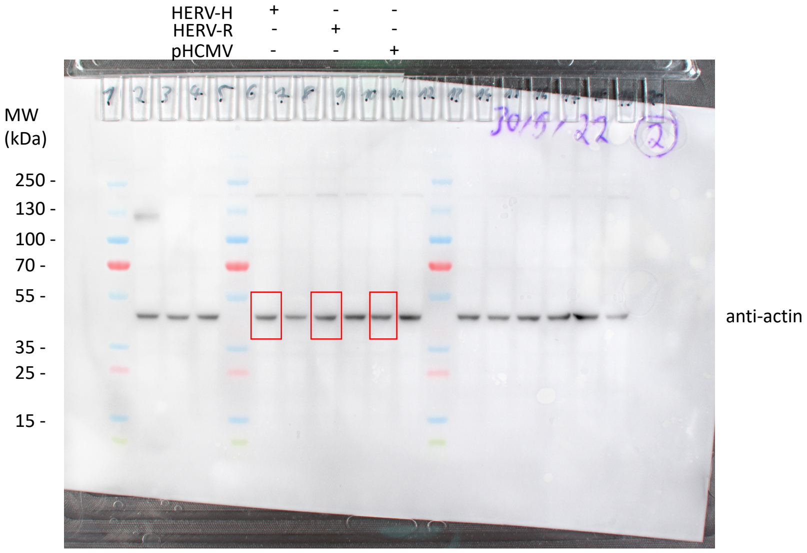

Supplementary Fig. 9f
Do. HEK NM-HAagg

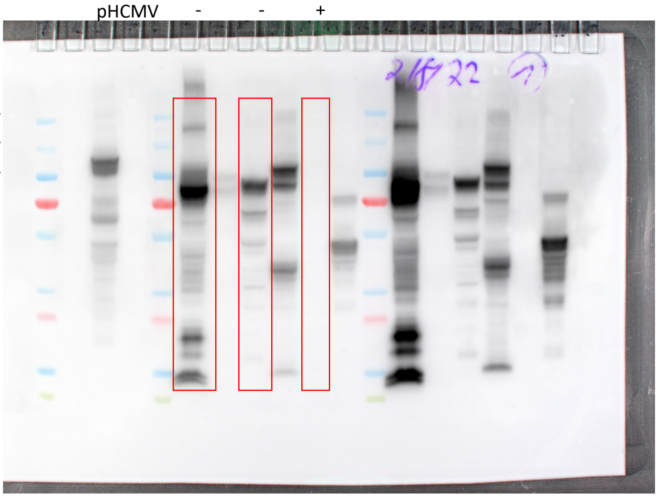

Supplementary Fig. 9f
Do. HEK NM-HAagg

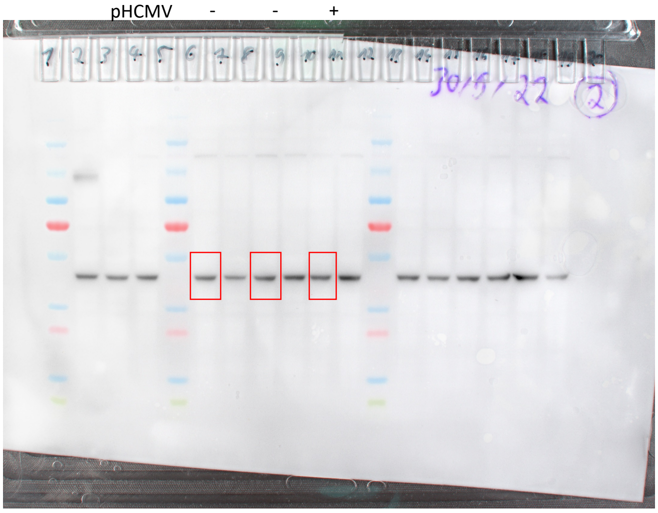

Supplement: Supplementary file 1 — Supplementary Information [file 41467_2023_40632_MOESM1_ESM.pdf]
