# Supplementary material for: Reactivated endogenous retroviruses promote protein aggregate spreading
Source: Nat Commun. 2023 Aug 18;14:5034. doi: 10.1038/s41467-023-40632-z (PMC10439213; doi:10.1038/s41467-023-40632-z)
Supplement: Supplementary file 3 — Description of Additional Supplementary Files [file 41467_2023_40632_MOESM3_ESM.pdf]

**Description of Additional Supplementary Files**

File Name: Supplementary Data 1
Description: Proteomic results of cell lysates comparing P16 and P07, and proteomic results of extracellular vesicle comparing P15 and P06.
