# Supplementary material for: Reactivated endogenous retroviruses promote protein aggregate spreading
Source: Nat Commun. 2023 Aug 18;14:5034. doi: 10.1038/s41467-023-40632-z (PMC10439213; doi:10.1038/s41467-023-40632-z)
Supplement: Supplementary file 5 — Reporting Summary [file 41467_2023_40632_MOESM5_ESM.pdf]

nature portfolio

Corresponding author(s): Ina Vorberg
Last updated by author(s): 14.07.2023

Reporting Summary

Nature Portfolio wishes to improve the reproducibility of the work that we publish. This form provides structure for consistency and transparency in reporting. For further information on Nature Portfolio policies, see our Editorial Policies and the Editorial Policy Checklist.

Statistics

For all statistical analyses, confirm that the following items are present in the figure legend, table legend, main text, or Methods section.

n/a	Confirmed	
☐	☒	The exact sample size (*n*) for each experimental group/condition, given as a discrete number and unit of measurement
☐	☒	A statement on whether measurements were taken from distinct samples or whether the same sample was measured repeatedly
☐	☒	The statistical test(s) used AND whether they are one- or two-sided<br>*Only common tests should be described solely by name; describe more complex techniques in the Methods section.*
☒	☐	A description of all covariates tested
☐	☒	A description of any assumptions or corrections, such as tests of normality and adjustment for multiple comparisons
☐	☒	A full description of the statistical parameters including central tendency (e.g. means) or other basic estimates (e.g. regression coefficient) AND variation (e.g. standard deviation) or associated estimates of uncertainty (e.g. confidence intervals)
☐	☒	For null hypothesis testing, the test statistic (e.g. *F*, *t*, *r*) with confidence intervals, effect sizes, degrees of freedom and *P* value noted<br>*Give P values as exact values whenever suitable.*
☒	☐	For Bayesian analysis, information on the choice of priors and Markov chain Monte Carlo settings
☒	☐	For hierarchical and complex designs, identification of the appropriate level for tests and full reporting of outcomes
☒	☐	Estimates of effect sizes (e.g. Cohen's *d*, Pearson's *r*), indicating how they were calculated

Our web collection on statistics for biologists contains articles on many of the points above.

Software and code

Policy information about availability of computer code

Data collection	Automated confocal microscope Cellvoyager CV6000 (Yokogawa Inc.)<br>Q-Exactive mass spectrometer (Thermo Scientific, US)<br>JEOL JEM-2200FS transmission electron microscope at 200 kV (JEOL)<br>Confocal laser scanning microscope LSM800 with Airyscan (Zeiss)<br>Imaging system Fusion FX (Evolution-Capt. Edge Ink, Vilber Lourmat)<br>Plate reader Fluostar Omega BMG (BMG Labtech)<br>ZetaView PMX 110-SZ-488 Nano Particle Tracking Analyzer (Measurement and Automation.Ink, Particle Metrix GmBH)
Data analysis	Automated image analysis was performed with Cellvoyager Analysis support software (Yokogawa Inc.: CV7000 Analysis Software; Version 3.5.1.18)<br>Confocal microscope images were analyzed via Zen 2010 (3.1, Blue Edition, ZenBlue, Zeiss)<br>Plate reader data was analyzed using MARS Data Analysis Software (Version: 3.40 R2, BMG Labtech)<br>Western Blot quantifications were done using Image J (Version 2.00-rc-69/1.52p)<br>MassSpec data were analyzed using Maxquant Software (maxquant.org, Max Planck Institute Munich, Version 1.5.5.1)<br>Statistical analyses were performed using Prism 6.0 (GraphPad Software v.7.0c).

For manuscripts utilizing custom algorithms or software that are central to the research but not yet described in published literature, software must be made available to editors and reviewers. We strongly encourage code deposition in a community repository (e.g. GitHub). See the Nature Portfolio guidelines for submitting code & software for further information.

Data

Policy information about availability of data

All manuscripts must include a data availability statement. This statement should provide the following information, where applicable:

- Accession codes, unique identifiers, or web links for publicly available datasets
- A description of any restrictions on data availability
- For clinical datasets or third party data, please ensure that the statement adheres to our policy

The mass spectrometry proteomics data generated in this study (Figure 1h and i) are available via the ProteomeXchange Consortium through the PRIDE partner repository under the accession code PXD043201. The following vector plasmids are deposited on Addgene for distribution (http://www.addgene.org) #15802, # 15805, # 35614, # 17576# 12253# 12259# 12251. The MS data was searched against a FASTA database of Mus musculus from UniProt including also non-reviewed entries supplemented with databases of lentiviruses and murine leukemia viruses (download: December 09th 2017, 52041 + 712 + 43 entries, https://www.uniprot.org/). Sequence data used for plasmid generation can be accessed under accession codes: AY037928; CAB94193.2; Q9N2K0.1; AAD34324.1; AAA88027.1 and AAF28334. The authors declare, that the remaining raw data or sequence information supporting the findings of this study are provided within the manuscript, methods section, its Supplementary Information or Source Data file and any other raw data that is not mentioned and that may be necessary to describe our work
will be made available upon request.. Source data are provided with this paper.

Research involving human participants, their data, or biological material

Policy information about studies with human participants or human data. See also policy information about sex, gender (identity/presentation), and sexual orientation and race, ethnicity and racism.

Reporting on sex and gender	not applicable
Reporting on race, ethnicity, or other socially relevant groupings	not applicable
Population characteristics	not applicable
Recruitment	not applicable
Ethics oversight	not applicable

Note that full information on the approval of the study protocol must also be provided in the manuscript.

Field-specific reporting

Please select the one below that is the best fit for your research. If you are not sure, read the appropriate sections before making your selection.

☒ Life sciences ☐ Behavioural & social sciences ☐ Ecological, evolutionary & environmental sciences

For a reference copy of the document with all sections, see nature.com/documents/nr-reporting-summary-flat.pdf

Life sciences study design

All studies must disclose on these points even when the disclosure is negative.

Sample size	Sample size was determined based on empirical data from the existing publications and experience in similar studies performed previously by our laboratory and the DZNE laboratory automation facility (Duernberger et al. 2018, doi: 10.1128/MCB.00111-18).
Data exclusions	No data was excluded from analyses.
Replication	6 wells with cells were analyzed per coculture experiment (n=6). 3 wells were analyzed per EV experiment (n=3). For quantitative analysis, at least 6000 cells were analyzed. Experiments were repeated at least twice, with similar results, except for experiments with conditioned medium of donors transfected with MLV gag/pol and env constructs (Fig. 6p). Two independent experiments, with similar results, with quantitative analysis performed after 4h and 24h.
Randomization	Allocation of cell culture populations cocultured or exposed to extracellular vesicles (EV) obtained from target cells or EV obtained from control cells treated with inhibitors, lentiviral infections, and transfections were randomly administered to cell populations that were otherwise cultured identically prior to the experiments.
Blinding	No blinding was done. Automated image acquisition and analysis was performed to minimize bias

Behavioural & social sciences study design

All studies must disclose on these points even when the disclosure is negative.

Study description	not applicable
Research sample	not applicable
Sampling strategy	not applicable
Data collection	not applicable
Timing	not applicable
Data exclusions	not applicable
Non-participation	not applicable
Randomization	not applicable

Ecological, evolutionary & environmental sciences study design

All studies must disclose on these points even when the disclosure is negative.

Study description	not applicable
Research sample	not applicable
Sampling strategy	not applicable
Data collection	not applicable
Timing and spatial scale	not applicable
Data exclusions	not applicable
Reproducibility	not applicable
Randomization	not applicable
Blinding	not applicable

Did the study involve field work? ☐ Yes ☒ No

Field work, collection and transport

Field conditions	not applicable
Location	not applicable
Access & import/export	not applicable
Disturbance	not applicable

Reporting for specific materials, systems and methods

We require information from authors about some types of materials, experimental systems and methods used in many studies. Here, indicate whether each material, system or method listed is relevant to your study. If you are not sure if a list item applies to your research, read the appropriate section before selecting a response.

Materials & experimental systems

n/a	Involved in the study
☐	☒ Antibodies
☐	☒ Eukaryotic cell lines
☒	☐ Palaeontology and archaeology
☒	☐ Animals and other organisms
☒	☐ Clinical data
☒	☐ Dual use research of concern
☒	☐ Plants

Methods

n/a	Involved in the study
☒	☐ ChIP-seq
☒	☐ Flow cytometry
☒	☐ MRI-based neuroimaging

Antibodies

Antibodies used

rat hybridoma anti-MLV Env (mAb83A25, 1:10; kindly provided by L.H. Evans, Rocky Mountain Laboratories, MT)
goat anti-xenotropic MLV virus antibody (antibodies-online, ABIN457298, 1:1000)
mouse anti-MLV gag (Abcam, ab100970, 1:1000)
rat anti-HA (Roche, 3F10, lot: 42155800, 1:1000)
mouse anti-GAPDH 6C5 (Abcam, ab8245, lot: GR3275542-4, 1:5000)
mouse anti-Actin C4 (MP Biomedical, 691001 , 1:5000)
rabbit anti-Tau (Abcam, ab64193, lot: GR3275542-1, 1:1000)
mouse anti PiT-2 (Santa Cruz Biotechnology, B-4, sc-377326, 1:1000)
rabbit anti-V5 (Cell signaling, D3H8Q, Lot: 6, 1:1000)
mouse anti-Alix (BD Bioscience, 611620, lot: 6217567, 1:1000)
mouse anti-Hsc/Hsp70 (ENZO, N27F3-4, lot: 09061121, 1:1000)
rabbit anti-Flotillin1 (Abcam, ab 133497 , lot: GR217473-1, 1:1000)
Alexa Fluor 647 goat anti-rat IgG (Invitrogen, A21247, lot: 1858181, 1:800)
anti-mouse horseradish peroxidase (HRP)-conjugated secondary AB (Dianova, 115-035-003; 1:10.000)
anti-rat HRP-conjugated secondary AB (Dianova, 112-035-003; 1:10.000)
anti-rabbit HRP-conjugated secondary AB (Dianova, 111-035-003; 1:10.000)

Validation

Commercially available antibodies were used according to the data sheets provided by the manufacturers for each assay.
Validation:Anti-MLV Env (rat hybridoma, mAb83A25) was generated and validated by Evans et. Al., Rocky Mountain Laboratories, MT). The details can be found in the following article: Evans LH, Morrison RP, alik FG, Portis J, Britt WJ. A. A neutralizable epitope common to the envelope glycoproteins of ecotropic, polytropic, xenotropic, and amphotropic murine leukemia viruses. J Virol 64, 6176-6183 (1990).
Goat anti-xenotropic MLV virus antibody (antibodies-online, ABIN457298). In-house validation by WB, detecting appropriate size protein. https://www.citeab.com/antibodies/2676035-abin457298-anti-xenotropic-murine-leukemia-virus-gp7
Mouse anti-MLV gag (Abcam, ab100970). Suitable for WB, ELISA. Reacts with murine leukemia virus GAG p30 capsid protein. 6 references provided by supplier (Abcam). In-house validation by WB, detecting appropriate size protein. https://www.abcam.com/products/primary-antibodies/gag-antibody-ab100970.html
rat anti-HA (Roche, 3F10, lot: 42155800). In-house validation by WB of ectopically expressed HA-tagged proteins. https://www.sigmaaldrich.com/DE/de/product/roche/12158167001
mouse anti-GAPDH (Abcam, ab8245, clone 6C5 , lot :GR3275542-4). Suitable for WB, ICC/IF (2735 references provided by Abcam). Reacts with mouse, rat, human. In-house validation by WB, detecting appropriate size protein. https://www.abcam.com/products/primary-antibodies/gapdh-antibody-6c5-loading-control-ab8245.html
Mouse anti-Actin (MP Biomedical, 691001, C4). Reacts with human, mouse, rat and guinea pig. Suitable for WB, ICC/IF. In-house validation by WB detecting correct size protein. https://www.mpbio.com/bs/anti-actin-mouse-monoclonal-antibody-clone-c4
rabbit anti-Tau (Abcam, ab64193, lot: GR3244995-1). Suitable for WB, ICC. Reacts with mouse, zebrafish. 55 references provided by supplier (Abcam). In house validation WB of ectopically expressed Tau. https://www.citeab.com/antibodies/753638-ab64193-anti-tau-antibody
Mouse anti-PiT-2 (Santa Cruz Biotechnology, sc-377326). Suitable for WB, ICC/IF, ELISA, IP. (9 references provided by Santa Cruz Biotechnology). Reacts with mouse, rat, human. In-house validation by WB, detecting appropriate size protein. https://www.scbt.com/de/p/pit2-antibody-b-4
Rabbit anti-V5 (Cell Signaling, D3H8Q, Lot: 6). In-house validation by WB of ectopically expressed V5-tagged proteins. https://www.cellsignal.com/products/primary-antibodies/v5-tag-d3h8q-rabbit-mab/13202
mouse anti-Alix (BD Bioscience, 611620, lot: 6217567). In-house validation by WB, detecting appropriate size protein. https://www.bdbiosciences.com/en-br/products/reagents/western-blotting-and-molecular-reagents/western-blot-reagents/purified-mouse-anti-aip1.611620
mouse anti-Hsc/Hsp70 (ENZO, N27F3-4, lot 09061121). Reacts with human, mouse, rat and others. Suitable for Flow Cytometry, IHC (PS), IP, WB. In-house validation by WB detecting correct size protein. https://www.enzolifesciences.com/ADI-SPA-820/hsc70-hsp70-monoclonal-antibody-n27f3-4/
rabbit anti-Flotillin1 (Abcam, ab133497, clone EPR6041, lot: GR217473-1). Suitable for WB, IHC-P, IP, ICC/IF. Knock-out validated. Reacts with mouse, rat, human. 23 references provided by Abcam). In-house validated by WB, detecting appropriate size protein. https://www.abcam.com/products/primary-antibodies/flotillin-1-antibody-epr6041-ab133497.html
Uncropped gel images are provided with the manuscript source data.

Eukaryotic cell lines

Policy information about cell lines and Sex and Gender in Research

Cell line source(s)	HEK 293T and Neuro-2a cells were purchased from ATCC. Vero cells were purchased from CLS (Cell lines service). Human astrocytes (Cat No.: 1800; Lot No.: 25536) were purchased from ScienCell Research Laboratories. Melan-a cells were purchased from Wellcome Trust, UK.
Authentication	All cell lines from ATCC, CLS and Wellcome Trust were authenticated via Short tandom repeat (STR) profiling.
Mycoplasma contamination	All cell lines were regularly tested for mycoplasma using PCR Mycoplasma Test Kit II A8994 (PanReac AppliChem), lot 11151118. Cell lines were free of mycoplasma contamination.
Commonly misidentified lines (See ICLAC register)	None.

Palaeontology and Archaeology

Specimen provenance	not applicable
Specimen deposition	not applicable
Dating methods	not applicable

☐ Tick this box to confirm that the raw and calibrated dates are available in the paper or in Supplementary Information.

Ethics oversight	not applicable

Note that full information on the approval of the study protocol must also be provided in the manuscript.

Animals and other research organisms

Policy information about studies involving animals; ARRIVE guidelines recommended for reporting animal research, and Sex and Gender in Research

Laboratory animals	RjOrl:SWISS Mus musculus outbread (Janvier-Labs, France).
Wild animals	not applicable
Reporting on sex	Sex was not considered for preparation of cortical neuronal cultures.
Field-collected samples	not applicable
Ethics oversight	SWISS Mice were housed and handled according to standards of the German Animal Welfare Act (23 °C, 40–50 % humidity, ad libitum access to food and water) with a 12 hours light/dark cycle. Mice were euthanized according to the German Animal Welfare Act for organ harvest according to § 4 Abs. 3 TierSchG. Since this procedure only needs to be notified, there is no protocol number.

Note that full information on the approval of the study protocol must also be provided in the manuscript.

Clinical data

Policy information about clinical studies
All manuscripts should comply with the ICMJE guidelines for publication of clinical research and a completed CONSORT checklist must be included with all submissions.

Clinical trial registration	not applicable
Study protocol	not applicable
Data collection	not applicable
Outcomes	not applicable

Dual use research of concern

Policy information about dual use research of concern

Hazards

Could the accidental, deliberate or reckless misuse of agents or technologies generated in the work, or the application of information presented in the manuscript, pose a threat to:

No	Yes	
☒	☐	Public health
☒	☐	National security
☒	☐	Crops and/or livestock
☒	☐	Ecosystems
☒	☐	Any other significant area

Experiments of concern

Does the work involve any of these experiments of concern:

No	Yes	
☒	☐	Demonstrate how to render a vaccine ineffective
☒	☐	Confer resistance to therapeutically useful antibiotics or antiviral agents
☒	☐	Enhance the virulence of a pathogen or render a nonpathogen virulent
☒	☐	Increase transmissibility of a pathogen
☒	☐	Alter the host range of a pathogen
☒	☐	Enable evasion of diagnostic/detection modalities
☒	☐	Enable the weaponization of a biological agent or toxin
☒	☐	Any other potentially harmful combination of experiments and agents

Plants

Seed stocks	not applicable
Novel plant genotypes	not applicable
Authentication	not applicable

ChIP-seq

Data deposition

☐ Confirm that both raw and final processed data have been deposited in a public database such as GEO.

☐ Confirm that you have deposited or provided access to graph files (e.g. BED files) for the called peaks.

Data access links<br>*May remain private before publication.*	not applicable
Files in database submission	not applicable
Genome browser session<br>(e.g. UCSC)	not applicable

Methodology

Replicates	not applicable
Sequencing depth	not applicable
Antibodies	not applicable

Peak calling parameters	not applicable
Data quality	not applicable
Software	not applicable

Flow Cytometry

Plots

Confirm that:

☐ The axis labels state the marker and fluorochrome used (e.g. CD4-FITC).

☐ The axis scales are clearly visible. Include numbers along axes only for bottom left plot of group (a 'group' is an analysis of identical markers).

☐ All plots are contour plots with outliers or pseudocolor plots.

☐ A numerical value for number of cells or percentage (with statistics) is provided.

Methodology

Sample preparation	not applicable
Instrument	not applicable
Software	not applicable
Cell population abundance	not applicable
Gating strategy	not applicable

☐ Tick this box to confirm that a figure exemplifying the gating strategy is provided in the Supplementary Information.

Magnetic resonance imaging

Experimental design

Design type	not applicable
Design specifications	not applicable
Behavioral performance measures	not applicable

Acquisition

Imaging type(s)	not applicable
Field strength	not applicable
Sequence & imaging parameters	not applicable
Area of acquisition	not applicable

Diffusion MRI ☐ Used ☒ Not used

Preprocessing

Preprocessing software	not applicable
Normalization	not applicable
Normalization template	not applicable
Noise and artifact removal	not applicable
Volume censoring	not applicable

Statistical modeling & inference

Model type and settings: not applicable

Effect(s) tested: not applicable

Specify type of analysis: ☐ Whole brain ☐ ROI-based ☐ Both

Statistic type for inference: not applicable

(See Eklund et al. 2016)

Correction: not applicable

Models & analysis

n/a	Involved in the study
☒	☐ Functional and/or effective connectivity
☒	☐ Graph analysis
☒	☐ Multivariate modeling or predictive analysis

Functional and/or effective connectivity: not applicable

Graph analysis: not applicable

Multivariate modeling and predictive analysis: not applicable
